# Assessment of Chicken Carcass Microbiome Responses During Processing in the Presence of Commercial Antimicrobials Using a Next Generation Sequencing Approach

**DOI:** 10.1038/srep43354

**Published:** 2017-02-23

**Authors:** Sun Ae Kim, Si Hong Park, Sang In Lee, Casey M. Owens, Steven C. Ricke

**Affiliations:** 1 Center for Food Safety, Department of Food Science, University of Arkansas, Fayetteville, AR 72704 USA; 2 Department of Poultry Science, University of Arkansas, Fayetteville, AR 72701 USA

## Abstract

The purpose of this study was to 1) identify microbial compositional changes on chicken carcasses during processing, 2) determine the antimicrobial efficacy of peracetic acid (PAA) and Amplon (blend of sulfuric acid and sodium sulfate) at a poultry processing pilot plant scale, and 3) compare microbial communities between chicken carcass rinsates and recovered bacteria from media. Birds were collected from each processing step and rinsates were applied to estimate aerobic plate count (APC) and *Campylobacter* as well as *Salmonella* prevalence. Microbiome sequencing was utilized to identify microbial population changes over processing and antimicrobial treatments. Only the PAA treatment exhibited significant reduction of APC at the post chilling step while both Amplon and PAA yielded detectable *Campylobacter* reductions at all steps. Based on microbiome sequencing, Firmicutes were the predominant bacterial group at the phyla level with over 50% frequency in all steps while the relative abundance of Proteobacteria decreased as processing progressed. Overall microbiota between rinsate and APC plate microbial populations revealed generally similar patterns at the phyla level but they were different at the genus level. Both antimicrobials appeared to be effective on reducing problematic bacteria and microbiome can be utilized to identify optimal indicator microorganisms for enhancing product quality.

Chickens and other poultry products are some of the most popular primary food products throughout the world^1^. However, poultry products can be contaminated by pathogenic bacteria such as *Salmonella* and *Campylobacter* thus their presence has been frequently implicated in outbreaks associated with consumption of poultry products^2^,^3^,^4^. As consumers become more interested in food safety and the consumption of poultry and poultry products increase, contamination of those bacteria is a major concern of poultry related industries, consumers, and government agencies such as US Department of Agriculture (USDA) and the Food Safety and Inspection Service^5^,^6^. Thus, it is important to develop effective interventions which can be applicable to poultry processing to insure microbiological safety^7^,^8^.

Chlorine has traditionally been used as an antimicrobial treatment during poultry processing and various alternative antimicrobial treatments have also been utilized to reduce pathogenic bacteria contamination including acidified sodium chlorite, cetylpyridinium chloride, chlorine dioxide, gamma irradiation, ozone, sodium hypochlorite, and trisodium phosphate^9^,^10^,^11^,^12^,^13^,^14^,^15^. However, the practical use of most of these antimicrobial treatments is limited due to the chemical residues having potential adverse effects to human, discoloration of chicken, avoidance by the consumer, corrosiveness to equipment, high cost, or limited effectiveness^2^,^16^.

Peracetic acid (PAA), a mixture of acetic acid and hydrogen peroxide, has been used as an antimicrobial in the food and poultry industries since PAA rapidly decomposes to acetic acid, oxygen, and water without formation of toxic residues, it can be easily applied (in water solution), and it is also economical due to its relatively low cost^17^. The use of PAA in poultry has been approved by the U.S. Food and Drug Administration (FDA) (21 CFR 173.370). A proprietary blend of surfuric acid and sodium sulfate is also commercially available (Amplon, Zoetis, Florham Park, NJ) as an antimicrobial to control bacterial contamination on poultry products and it also possesses economic and environmental benefits^8^. Amplon is comprised of ingredients which are classified as generally recognized as safe (GRAS) by FDA and is also an approved processing aid and antimicrobial by the USDA (FSIS 7120.1) for poultry use as a spray, wash or dip thus its application is feasible in the poultry industry. When Amplon was applied to chicken wings inoculated with *Salmonella* at pH 1.1 for 10 or 20 s, Amplon exhibited significant antimicrobial activities (pathogen reduction of 0.8–1.2 log CFU/ml) and its efficacy was higher than that of cetylpyridinium chloride which is commonly used by the poultry industry^8^. However, to the best of our knowledge, no studies have examined the empirical antimicrobial activities of Amplon with whole chicken carcasses on a pilot plant scale.

To date, the microbiological analysis of indicator organisms during the general chicken processing or the efficacy of antimicrobial treatment in reducing *Campylobacter* or *Salmonella* on chicken carcasses has been the focus of most research efforts^2^,^11^,^18^,^19^. However, there have only been limited studies focused on microbiome and microbial communities on whole chicken along with general chicken processing steps as well as before and after antimicrobial treatments. To improve the microbiological safety of chicken products, more information is needed on how carcass bacterial associated communities are altered during processing and which poultry-associated bacteria (both beneficial and harmful) are reduced or retained during the application of processing by steps or treatments. Documenting how microbial community changes from a phylum level to a genus level may help achieve in-depth understanding of the microbial dynamics during processing steps and/or antimicrobial treatments, to predict potential microbiological hazards, and to better understand responses of the pathogens or spoilage bacteria present on these products. High-throughput next generation sequencing (NGS) platforms which are based on 16S rRNA gene amplicons have mostly been utilized to specifically address the complex aspects of the gastrointestinal microbiome in animals as well as humans but offers utilities for postharvest applications as well^20^,^21^,^22^,^23^,^24^,^25^.

In the present study, we investigated the population recovered from aerobic plate count petrifilm (APCs) and *Campylobacter* as well as *Salmonella* prevalence along with general chicken processing steps by examining samples taken at different stages of the first manufacturing process. Also, antimicrobial treatments at four different locations using PAA and Amplon (Amplon spray after depilation step, on-line reprocessing with Amplon, post-chilling with PAA, and post-chilling with Amplon) were applied during chicken processing and their antimicrobial activities against APCs, *Campylobacter*, and *Salmonella* were evaluated from chicken carcass rinsate samples. Secondly, an Illumina MiSeq was utilized to not only perform microbiome sequencing on chicken carcass rinsates but of all colonies present on 3M APC petrifilm and *Campylobacter* selective media in parallel with the rinsate samples to identify recovered colony microbiota via sequencing and assess how traditional plating matched the microbial composition of the rinsates.

## Results

### Microbiological analysis

#### General chicken processing step

**Figure 1:**
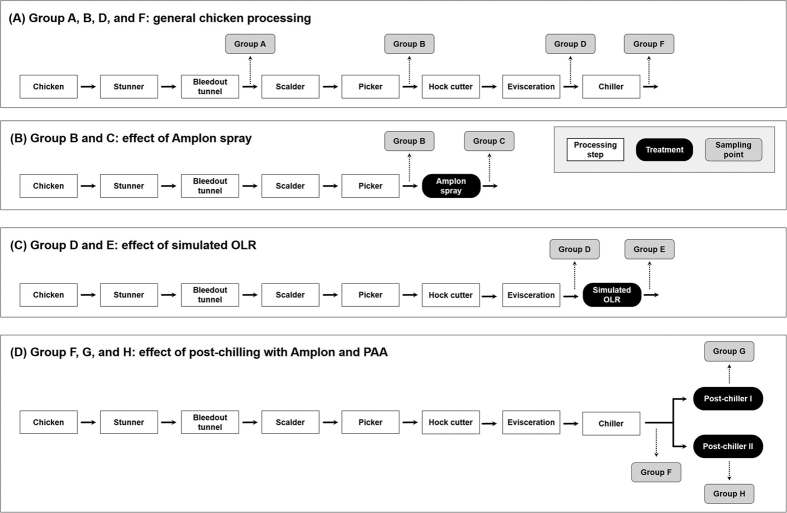
Diagram to illustrate the first processing stage for chicken carcass, antimicrobial treatments, and the sampling points taken for microbial analyses and microbiome. Condition of antimicrobial treatment: Amplon spray (pH 1.3); simulated on-line reprocessing (OLR) with Amplon (pH 1.4 and a 15 s dip); post-chilling with Amplon (pH 1.4 and 15 s dip); post-chilling with PAA (750 ppm and 15 s dip). Ten birds were taken from each group (total 80 birds). PAA, peracetic acid.

**Figure 2:**
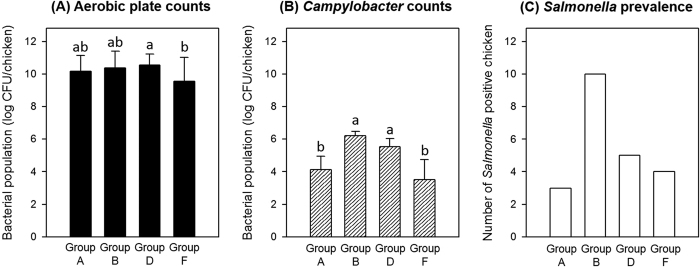
Average populations of aerobic plate counts (**A**) and *Campylobacter* (**B**) and *Salmonella* prevalence (**C**) on chicken carcasses (n = 10, each group) during general chicken processing steps. Values denoted by the same letter within each microbial group were not significantly different. All counts were considered significantly different at *P* < 0.05.

Poultry processing stages and all sampling points are shown in Fig. 1A–D. Group A and processing steps prior to antimicrobial addition (groups B, D, and F) represent typical poultry processing steps (Fig. 1A). Recovered APCs and *Campylobacter* populations as well as *Salmonella* prevalence in the chicken carcasses at each processing step are shown in Fig. 2. Quantitative contamination levels of APCs and *Campylobacter* were analyzed and qualitative prevalence levels of *Salmonella* were investigated. Quantitative data were expressed as log CFU/chicken to present the corresponding bacterial populations obtained from whole chicken carcasses. Average APCs of the initial group (group A, after bleed out) was 10.16 log CFU/chicken. The APCs were maintained after scalder, picker (group B, 10.37 log CFU/chicken), hock cutter, and evisceration (group D, 10.55 log CFU/chicken) (*P* > 0.05) and they were slightly reduced by the chilling step (group F, 9.92 log CFU/chicken) (*P* < 0.05). For *Campylobacter* populations, group A yielded 4.14 log CFU/chicken and these levels were significantly increased to 6.20 log CFU/chicken in the group B (*P* < 0.05) followed by consistent incremental reduction to 5.55 log CFU/chicken in the group D to 3.50 log CFU/chicken in the group F. For *Salmonella* prevalence, the percent of chicken exhibiting *Salmonella* positive was 30% for group A, 100% for group B, 50% for group D, and 40% for group F.

### Effects of antimicrobial treatments

**Figure 3:**
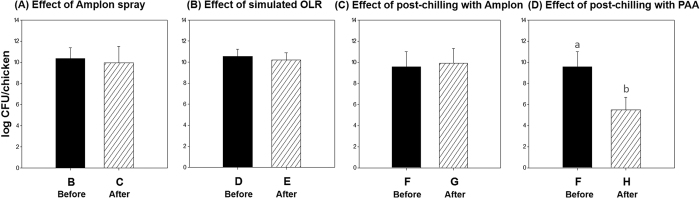
Bacterial reduction of aerobic plate counts on chicken carcasses (n = 10, each group) by antimicrobial treatments including Amplon spray, simulated OLR, post-chilling with Amplon, and post-chilling with PAA. Values denoted by the same letter within each microbial group were not significantly different. All counts were considered significantly different at *P* < 0.05. PAA, peracetic acid.

Different antimicrobial treatment at four locations including 1) Amplon spray (before: group B, after: group C), 2) simulated OLR (before: group D, after: group E), 3) post-chilling with Amplon (before: group F, after: group G), and 4) post-chilling with PAA (before: group F, after: group H) were applied during chicken processing (Fig. 1B,C and D). Reduction of APCs in the chicken carcasses by antimicrobial treatments are presented in Fig. 3. There was no significant differences in recovered APCs (*P* > 0.05) between group B (10.37 log CFU/chicken) and C (9.95 log CFU/chicken) indicating Amplon spray did not affect the APC populations on chicken carcasses. Simulated OLR and post-chilling with Amplon exhibited similar results; there was no significant differences in APCs between before and after treatment. Only post-chilling with PAA resulted in a significant reduction (4.08 log CFU/chicken reduction) of APCs (*P* < 0.05). It appears that post-chilling with PAA can effectively limit APC levels on chicken carcasses.

**Figure 4:**
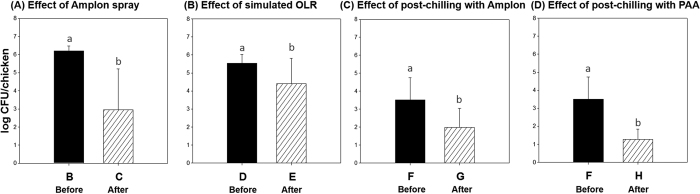
Bacterial reduction of *Campylobacter* populations on chicken carcasses (n = 10, each group) by antimicrobial treatments including Amplon spray, simulated OLR, post-chilling with Amplon, and post-chilling with PAA. Values denoted by the same letter within each microbial group were not significantly different. All counts were considered significantly different at *P* < 0.05. PAA, peracetic acid.

The reduction of *Campylobacter* populations in the chicken carcasses after antimicrobial treatment is shown in Fig. 4. Unlike the APC results, *Campylobacter* counts were significantly reduced by all antimicrobial treatments. The Amplon spray, simulated OLR with PAA, and post-chilling with Amplon or PAA resulted in 3.25, 1.15, 1.53, and 2.23 log CFU/chicken reductions, respectively (*P* < 0.05).

**Figure 5:**
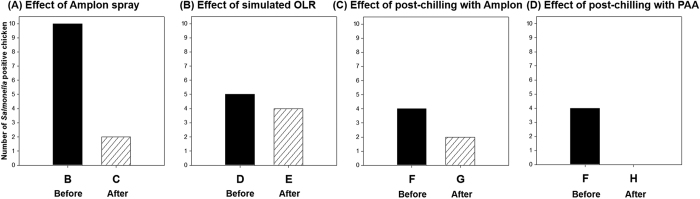
Reduction of number of *Salmonella* positive chicken carcasses (n = 10, each group) by antimicrobial treatments including Amplon spray, simulated OLR, post-chilling with Amplon, and post-chilling with PAA. PAA, peracetic acid.

The prevalence of *Salmonella* is shown in Fig. 5. The number of positive samples was markedly reduced by Amplon spray from 100% positive (group B) to 20% positive (group C). Simulated OLR rarely impacted *Salmonella* prevalence. Before and after simulated OLR samples yielded 50 (group D) and 40% (group E) positive carcasses, respectively. For post-chilling with Amplon, the number of positives was reduced from 40 (group F) to 20% positive (group G). When chicken carcasses were exposed to the post-chilling with PAA, *Salmonella* were not isolated from all birds after post-chilling (group H). The post-chilling with PAA was more effective than Amplon to reduce *Salmonella* prevalence.

### Microbiome analysis

#### Chicken rinsate: microbial correlation among groups

**Figure 6:**
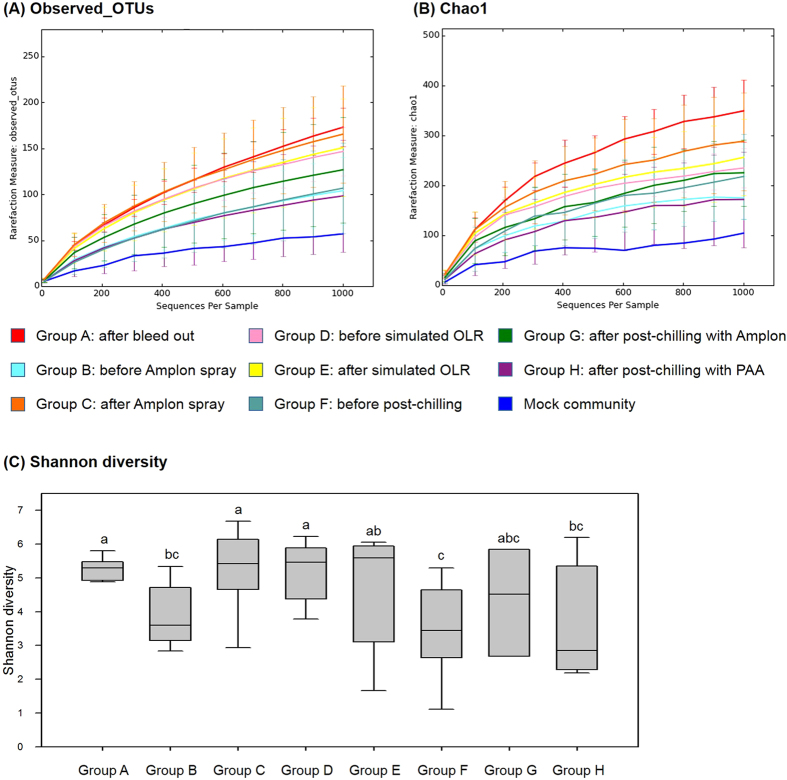
Alpha diversity analysis among groups. Rarefaction curves of (**A**) Observed_OTUs, (**B**) Chao 1, and (**C**) Shannon diversity. PAA, peracetic acid.

Each rarefaction of average observed_OTUs, Chao1 and Shannon diversity plots from alpha diversity analysis is shown in Fig. 6A–C respectively. As shown in Fig. 6A, earlier processing step samples exhibited greater observed OTU numbers compared to the other groups while group F (before post-chilling), G (post-chilling with Amplon), and H (post-chilling with PAA) yielded lower specific OTU numbers (Fig. 6A). The Chao 1 rarefaction plot was employed to estimate species richness and it revealed a similar result with the observed_OTU rarefaction plot (Fig. 6B). The Shannon diversity plot exhibited lower numbers in the later step samples (group F, G, and H) than earlier processing step samples (group A, C, D, and E) (Fig. 6C).

**Figure 7:**
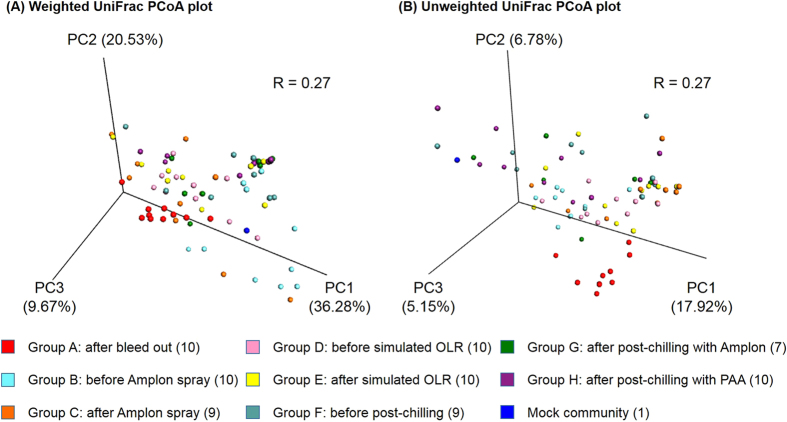
Beta diversity analysis among groups. (**A**) Weighted and (**B**) unweighted UniFrac PCoA plots of individual chickens in each group. PAA, peracetic acid.

Figure 7A and B represents weighted and unweighted principal coordinated analysis (PCoA) UniFrac plots generated by the beta diversity analysis, respectively. The weighted and unweighted PCoA UniFrac plots exhibited the relative abundance of OTUs among groups and their respective phylogenetic distances. In the weighted PCoA UniFrac plot, each data point representing an individual sample was aligned in parallel on the PC1 axis with 36.28%. An R value close to 1 was used to indicate that there was dissimilarity among groups while an R value near 0 meant no separation. An R value from both weighted and unweighted PCoA plots was 0.27 which implied no significant dissimilarity among groups. In both plots, only group A was distinctively clustered while detectable patterns of distinctive clustering were not observed in samples from groups B-H.

#### Chicken rinsate: bacterial OTUs abundance at the phylum level

**Figure 8:**
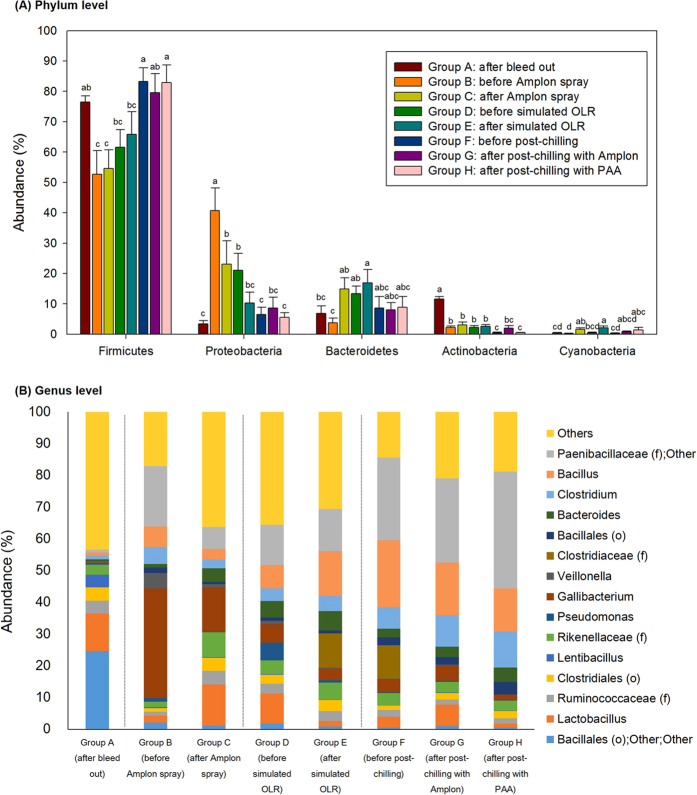
Relative abundance of major bacteria among different groups in chicken carcass rinsates at a phylum (**A**) and genus level (**B**). PAA, peracetic acid. f and o in parenthesis indicate family and order, respectively.

A total of 21 phyla were identified in the chicken rinsate. Figure 8A represents the top 5 bacterial phyla among groups identified in chicken rinsates. The chicken rinsates microbiota were dominated by Firmicutes (69.24% ± 2.44), Proteobacteria (15.15% ± 2.14), Bacteriodetes (10.19% ± 1.19), Actinobacteria (3.17% ± 0.45), and Cyanobacteria (0.95% ± 0.16) accounting for 98.70% of the entire phyla. Deferribacteres (0.22% ± 0.07), Tenericutes (0.20% ± 0.03), Synergistetes (0.2% ± 0.05), and Euryarchaeota (0.07% ± 0.02) were the other minor abundant phyla. Overall microbiota of each group revealed generally similar patterns; the top four dominant bacteria of all groups at the phylum level belonged to either Firmicutes, Proteobacteria, Bacteriodetes, or Actinobacteria, respectively (Fig. 8A).

However, their relative abundance at the phylum level was varied at different processing steps. Firmicutes was predominant for group A with 76.54% but their abundance was significantly decreased to 52.70% after the scalder and picker steps (group B) (*P* < 0.05). In the subsequent processing steps, Firmicutes abundance increased with the corresponding processing steps that included hock removal, evisceration (group D: 61.55%), and main chilling (group F: 83.24%). For Proteobacteria which was the second most predominant phylum, the relative abundance was much higher in group B (40.72%) as compared to group A (3.38%) and this group steadily declined as the processing progressed (group D: 21.09% and group F: 6.44%).

The Amplon spray (groups B and C), simulated OLR (groups D and E), post-chilling with Amplon (groups F and G), and post-chilling with PAA (groups F and H) did not affect Firmicutes abundance (*P* > 0.05). The Amplon spray and simulated OLR resulted in significant reductions in abundance of Proteobacteria (*P* < 0.05); 17.68% and 10.83% reduction, respectively, while their abundance was not changed by post-chilling with Amplon or PAA (*P* > 0.05).

#### Chicken rinsate: bacterial OTUs abundance at the genus level

Relative distributions of major OTUs in the chicken rinsates at the genus level are represented in Fig. 8B. A total of 280 OTUs were assigned to chicken rinsates. Bacterial composition of rinsates shifted during poultry processing. The top five predominant genera of group A representing the initial stage sample group in the processing cycle were Bacillales (order), *Lactobacillus, Ruminococcaceae* (family), Clostridiales (order), and *Lentibacillus* with a total of 48.74% but their abundance decreased in the following processing steps accounting for only 7.00 (group B), 17.45 (group D), and 7.47% (group F) (Fig. 8B), respectively. In contrast, *Gallibacterium, Clostridium, Bacillus*, and *Paenibacillaceae* (families) were only a small percentage of the total microbial communities in group A (3.34%) but they became more predominant in groups B, D, and F at 65.39, 29.82, and 58.01%, respectively.

For the Amplon spray treatment (groups B and C), there were significant decreases in abundance of *Gallibacterium* and *Paenibacillaceae* (family) from 34.54 to 14.10% and from 19.00 to 6.88%, respectively, while the abundance of *Lactobacillus* was increased from 2.09 to 13.01%. There was also a decrease in the abundance level of *Lactobacillus* (9.45 to 1.74%) and an increase of *Clostridiaceae* (family) (0.04 to 10.82%) by the simulated OLR treatment. Both post-chilling with Amplon and PAA resulted in significant reductions in *Clostridiaceae* (family) by 10.45 and 10.48%, respectively.

#### Aerobic petrifilm counts: bacterial OTUs abundance at the phylum level

**Figure 9:**
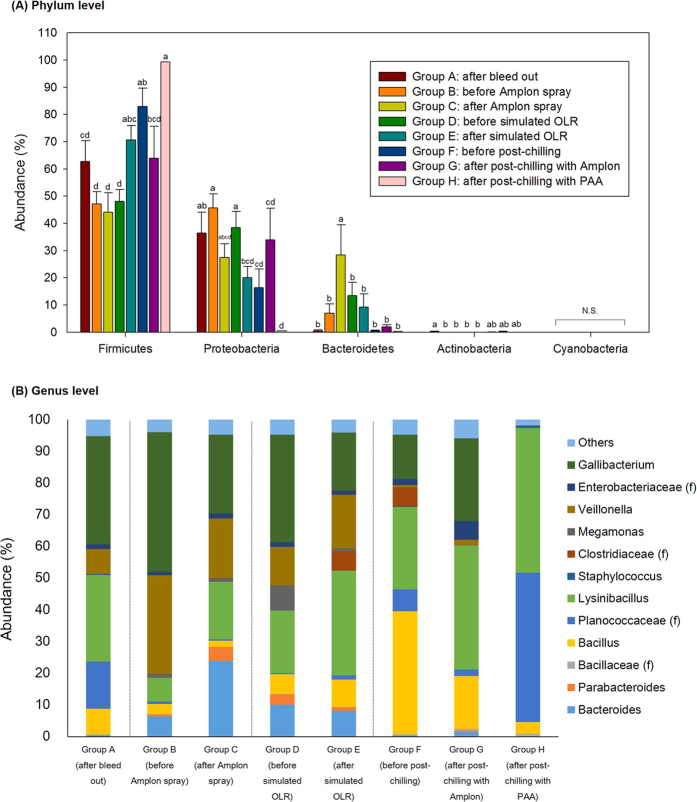
Relative abundance of major bacteria among different groups in APC Petrifilm at a phylum level (**A**) and genus level (**B**). PAA, peracetic acid. f in parenthesis indicate family.

Overall microbial distributions on APC petrifilm via sequencing revealed generally similar patterns compared to rinsate samples with some differences in minor groups. Most OTUs belonged to Firmicutes, Proteobacteria, and Bacteriodetes with 61.19% ± 2.99, 30.29% ± 2.70, and 8.39% ± 2.06 of the total phyla while Actinobacteria (0.07% ± 0.03) and Cyanobacteria (0.01% ± 0.00) were rarely detected in all groups (Fig. 9A). Similar to chicken rinsates results, the proportion of Firmicutes was generally increased (group B: 47.16%, group D: 48.05%, and group F: 63.92%) while that of Proteobacteria was decreased (group B: 45.73%, group D: 38.47%, and group F: 16.40%) along with subsequent processing steps.

The simulated OLR step resulted in the most significant increase in Firmicutes. The relative abundance of Firmicutes in group D was 48.05%; however, this group significantly increased to 70.61% in group E (*P* < 0.05). Post-chilling with Amplon caused a 19.08% reduction of Firmicutes while they were increased by 16.32% by post-chilling with PAA. For Proteobacteria, the abundance was highly reduced by Amplon spray (18.34%) and simulated OLR (18.44%).

#### Aerobic petrifilm counts: bacterial OTUs abundance at the genus level

At the genus level, a total 151 OTUs were identified. *Lysinibacillus* and *Gallibacterium* accounted for the highest abundance levels during general chicken processing steps with 61.52, 51.44, 53.61, and 39.94% in groups A, B, D, and F, respectively (Fig. 9B). The abundance level of *Bacillus* was significantly higher in group F (38.81%) compared to that of the other groups.

When antimicrobial treatments were applied, the abundance of *Lysinibacillus* was reduced considerably with 10.78, 13.31, 13.30, and 19.73% reduction by Amplon spray (groups B and C), simulated OLR (groups D and E), post-chilling with Amplon (groups F and G), and post-chilling with PAA (groups F and H), respectively. In contrast, *Gallibacterium* abundance was generally increased by antimicrobial treatments including 19.22, 15.59, and 13.99% increased by Amplon spray, simulated OLR, and post-chilling with PAA, respectively.

#### Colonies on Campy-Cefex selective media: bacterial OTUs abundance at the phylum level

**Figure 10:**
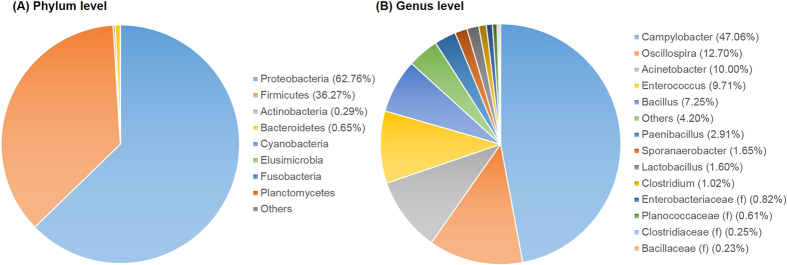
Relative abundance of major bacteria in colonies from Campy-Cefex selective media at a phylum level (**A**) and genus level (**B**). f in parenthesis indicate family.

In order to investigate the specificity of Campy-Cefex selective media, all colonies on the media were collected and sequenced. The distribution of bacteria from Campy-Cefex selective media among groups at the phylum level are shown in Fig. 10A. Firmicutes and Proteobacteria were relatively common while Bacteriodetes, Actinobacteria, and Cyanobacteria were much less abundant. Sequenced microbiota recovered from the Campy-Cefex selective media exhibited proportionally different phyla levels. The plates harbored the greatest proportion of Proteobacteria constituting 62.76% ± 4.30 of all phyla whereas Firmicute were most abundant in chicken rinsates and APC. This is likely due to the selective *Campylobacter* plates exclusively supporting growth of *Campylobacter* which belong to Proteobacteria.

#### Colonies on Campy-Cefex selective media: bacterial OTUs abundance at the genus level

Bacterial composition of major bacteria at the genus level are shown in Fig. 10B. Samples exhibited variable frequencies in the presence of the genus *Campylobacter*. The relative abundance of *Campylobacter* in groups A to G was 5.86, 64.78, 31.45, 82.01, 77.29, 49.79, and 18.11%, respectively (no bacteria were recovered in Campy-Cefex selective media from group H). Average abundance of *Campylobacter* was 47.06%. Other bacteria such as *Oscillospira* (12.70%), *Acinetobacter* (10.00%), *Enterococcus* (9.71%), *Bacillus* (7.25%), *Paenibacillus* (2.91%), *Sporanaerobacter* (1.65%), *Lactobacillus* (1.60%), and *Clostridium* (1.02%) were also apparently present on Campy-Cefex selective media.

## Discussion

Both *Salmonella* and *Campylobacter* are bacteria that can be detected on chicken carcasses^2^. Here, non-inoculated chickens in our study indicated that chicken carcasses had background *Campylobacter* and *Salmonella* at variable levels at the different processing steps. *Campylobacter* contamination levels (3.5 log CFU/chicken, Fig. 2) after the chilling step in this study is in agreement with those of previous studies showing *Campylobacter* populations of approximately 4,000 CFU/carcass as being typical^9^. On poultry carcasses, *Campylobacter* has been shown previously to be more prevalent than *Salmonella*^2^ and our studies also yielded similar results. *Campylobacter* populations in chicken carcass rinsates were recovered on an average of 3.75 log CFU/chicken (n = 80) based on standard selective media plating and abundance of *Campylobacter* in rinsates averaged 1.6% in microbiome analysis. In contrast, *Salmonella* was only detected by qualitative analysis which included an enrichment procedure and also was not detected in chicken carcass rinsates via microbiome sequencing.

In the present study, samples were taken directly from a pilot processing plant (from live bird to the final product) during a typical process similar to a commercial plant. Based on the results of microbiological characterization of the chicken rinsates, the major contamination route of *Campylobacter* and *Salmonella* appeared to be at the scalding or defeathering steps. *Campylobacter* populations and incidence of *Salmonella* were significantly increased between group A (before scalding and defeathering) and B (after scalding and defeathering) (Fig. 2). Group A represented poultry carcasses prior to depilation and head cutting. Since both *Campylobacter* and *Salmonella* are usually present in the intestinal tract, it is difficult to recover them when chicken carcasses still possess their heads^26^,^27^. Consequently a relatively low population (4.14 log CFU/chicken) of *Campylobacter* and detection level (30%) for *Salmonella* were recovered from group A (Fig. 2). After depilation and head removal (group B), chicken intestinal tracts were rinsed with buffer internally and externally in and out of the chicken carcasses thus resulting in the ability to detect *Campylobacter* (6.20 log CFU/chicken) and *Salmonella* (100% detection rate) on the chicken carcasses (Fig. 2). These results are in agreement with previous research that reported that populations of *Campylobacter* increased following the defeathering step^28^,^29^. Therefore, both foodborne pathogens probably originated from the intestinal tracks of the chickens. Consequently, hygienic management of scalding and defeathering steps as well as proper management of intestines could represent a critical control point to avoid cross-contamination of chicken meat. The chilling step is also a critical control point since it led to significant reduction of APC and *Campylobacter* counts and this is agreement with the common belief that the chilling step in poultry processing is the critical control step in reducing foodborne pathogens and spoilage bacteria^2^,^30^.

This study also evaluated antimicrobial activities of various treatments with 80 chicken carcasses on an actual processing line at the pilot plant to evaluate the stability and feasibility of antimicrobial treatments in a commercial plant. It is worthwhile to note that treatments (spraying, dipping and chilling) with both PAA and Amplon during the chicken processing plant operation were effective in reducing *Campylobacter* populations and the incidence of *Salmonella* (Figs 3 and 4). Our results demonstrated that post-chilling as well as other applications such as spraying and simulated OLR resulted in significant bacterial reductions. Also, one distinctive advantage of these technologies is that since the use of PAA and Amplon is officially approved in poultry processing, they already have commercial utility for immediate application in the commercial industry^8^,^17^. The present study should help to provide guidelines or recommendations to commercial poultry processing industries about intervention methods regarding optimal applications of PAA and Amplon.

Traditionally, detection and enumeration of bacteria in poultry at various stages including production, processing, distribution, or storage have focused on particular known bacteria such as *Campylobacter* and *Salmonella* and these studies were performed based on cultivation methodologies^24^. Currently, poultry microbiome analysis with high-throughput sequencing is of particular interest as a novel tool to assess bacterial communities associated with poultry and identify specific microorganisms since this tool can detect the microbiota census including non-cultivable organisms^24^. Most research studies employing microbiome sequencing technology for poultry have focused on gastrointestinal track microbiota^23^,^31^,^32^,^33^,^34^,^35^ and to the best of our knowledge, only limited efforts have focused on the chicken carcass microbiomes during processing. Oakley *et al*. (2013) applied high-throughput sequencing to a wide range of chicken samples including fecal, litter, carcass rinsates, and carcass weeps^24^. They collected chicken carcass rinses in the chlorinated chill tank. More recently, Rothrock *et al*.^30^ assessed the microbiome of scalder and chiller tank waters at a typical commercial poultry processing day but not the individual chicken carcass rinsates^30^. In this study, chicken carcass rinsates from individual birds at different processing step were subjected to microbiome sequencing.

The novelty of the present study is that microbiome analysis was conducted during typical chicken processing steps that included before/after antimicrobial treatments and comparing microbial populations from the rinsates as well as the culture plates. Identifying indigenous microbial communities and microbial dynamics throughout typical poultry processing steps by microbiome analysis should help to understand the microbial ecology of chicken carcasses. Here, the predominant abundance of Firmicute, Proteobacteria, Bacteroidetes, and Actinobacteria is consistent with the results for major poultry associated bacterial phyla at poultry production reported by Rothrock *et al*.^30^. The other interesting finding is that Proteobacteria abundance decreased as processing progressed. Proteobacteria are phylogenetically related to major foodborne pathogens; they include a wide range of foodborne pathogens including *Campylobacter, Salmonella, E. coli, Vibrio*, and *Yersinia*^36^,^37^,^38^.

Indicator bacteria have been used to evaluate both food quality and safety in the food industry thus finding the most appropriate indicator bacteria may play an important role to improve food quality and safety. Sequencing technology could provide a wide range of information to identify the appropriate indicator microorganisms during the manufacturing process. Also, this should help to better understand the microbial distribution of bacteria according to the presence or absence of specific foodborne pathogens. When bacterial abundances at the phylum level between *Salmonella* positive and negative chicken rinsates were compared, there were no significant differences in bacterial abundance in all phyla. At the genus level, *Salmonella* positive rinsate samples yielded a lower abundance of *Clostridium* compared with the *Salmonella* negative samples. In contrast, there was no significant differences in other predominant bacteria such as *Paenibacillaceae* (family), *Bacillus, Gallibacterium, Lactobacillus, Rikenellaceae* (family), Bacillales (order), *Bacteroides, Ruminococcaceae* (family), Bacillales (order), *Pseudomonas, Veillonella*, and *Lentibacillus*. When bacterial abundances between *Campylobacter* positive and negative chicken rinsates were compared at the phylum level, a higher abundance of Actinobacteria (3.74%) was exhibited in the *Campylobacter* positive rinsate than the *Campylobacter* negative sample (1.70%). At the genus level, greater abundances of *Paenibacillaceae* (family, 24.98%) and *Clostridium* (8.41%) were observed in *Campylobacter* negative samples compared with the positive samples accounting for 14.48% of *Paenibacillaceae* and 4.49% of *Clostridium*. However, Bacillales (order) exhibited lower levels in the *Campylobacter* negative rinsate (0.82%) than the positive samples (5.68%).

*Salmonella* are a representative foodborne pathogen group causing foodborne illness in humans and it is well known that one of their primary vehicles is poultry products thus *Salmonella* identification in poultry products is essential^39^,^40^. In this study, *Salmonella* was not detected in any of the microbiome data including rinsate, APCs, and *Campylobacter* plates from any of the chicken samples. However, *Salmonella* was occasionally isolated from qualitative microbial analysis after an enrichment and selective isolation procedure. These results indicate that *Salmonella* even when present was not quantitatively a dominant bacteria in chicken rinsates accounting for only negligible levels thus making it difficult to detect without including an enrichment procedure. Selective isolation including a proper enrichment step to increase *Salmonella* populations in the sample is required to isolate *Salmonella* in samples such as chicken carcass rinsates. The conventional method used in this study is based on enriching with nutrient broth, plating on to selective and differential agar medium (XLT4), and identifying presumptive colonies with PCR. This method required considerable time to complete the isolation procedure (about 4 to 5 days) thus it could be problematic for the poultry industry which needs more rapid inspection. Development of more rapid and reliable methods which could quantify very low levels of *Salmonella* populations in chicken rinsates would be a valuable further study.

The use of Campy-Cefex selective media is recommended by USDA and has been widely utilized to isolate *Campylobacter* selectively on media^41^. *Campylobacter* was not a predominant bacteria in rinsate and APC Petrifilm (Figs 8 and 9) based on microbiome sequencing but as would be expected was a predominant bacteria on Campy-Cefex selective media (Fig. 10). This indicated that while Campy-Cefex selective media can support growth of *Campylobacter* from a mixed background sample, a wide range of bacterial communities can also be recovered from these plates. Consequently, various bacteria such as *Clostridiaceae, Paenibacillus, Lactobacillus, Bacillaceae, Acinetobacter, Enterobacteriaceae, Bacillus, Planococcaceae, Clostridium, Enterococcus, Sporanaerobacter*, and *Oscillospira* were also identified in the microbiome analysis at varying percentages in the Campy-Cefex selective media (Fig. 10). Even though these bacteria do not produce similar colony morphology in Campy-Cefex selective agar, their growth could inhibit or mask growth of *Campylobacter* if their microbial populations were to exceed that of *Campylobacter* on selective plates. Several researchers have reported limited abilities of Campy-Cefex selective media including less inhibitory effects on the background bacteria, a low accuracy and selectivity in chicken carcass rinsates^42^,^43^. Therefore, preventing growth of non-*Campylobacter* bacteria in media may be needed to improve selectivity of Campy-Cefex selective media (for example, adding antibiotics to inhibit growth of non-*Campylobacter* bacteria). Additional research is needed to conduct similar studies with other *Campylobacter* selective media.

Petrifilm which consists of dry rehydratable film containing nutrients and/or antibiotics and dye in a gelling agent, has been extensively utilized to analyze bacteria as well as yeast and mold^44^. Various types of commercial Petrifilm are popular for educational and industrial applications since they provide time-saving, convenient, and reliable results with a high degree of accuracy^45^,^46^. Petrifilm media have been used in a wide range of fields and their use has also been approved for standard analytical methods^41^,^47^. To the best of our knowledge, there have been no attempts to extract DNA from Petrifilm directly thus the current study represents the first successful recovery of DNA extracted from APC Petrifilm for sequencing analyses. The average concentration of extracted DNA with our modification method was 32.05 ± 3.61 ng/μl with purity 1.69 ± 0.01 and it was a sufficient quantity to conduct microbiome analysis and successfully complete the analysis, suggesting that our modified method offers a reasonable DNA extraction approach from a matrix such as the Petrifilm gel. Thus, the present method has implications for related research fields or industries which typically use Petrifilm and could facilitate expanded use of Petrifilm in microbiological analysis as well as molecular based methodologies.

While conducting routine daily NGS microbiome analyses in poultry processing would not currently be cost effective (for example, $100.00 USD per sample, https://genohub.com/bioinformatics/24/microbiome-analysis), there are certain circumstances where such analyses could be of considerable merit. Potential targets for application include confirming consistent effectiveness when antimicrobial protocols are altered or new products introduced and identifying bacterial cross contamination sources in individual processing plants originating either from incoming flocks of birds or residual microbial populations in the plant environment. In addition, individual members of these microbial communities may also serve as ideal indicator organisms for predicting potential presence of foodborne pathogens. Finally, microbiome information could provide more detail on microbial communities on poultry carcasses for predicting spoilage and shelf life of these products. This data in turn would potentially complement conventional plating by providing an independent quality control test on how representative the plating is of changes in microbial communities occurring during processing. It is anticipated that as NGS costs decrease microbiome analysis during processing could become more routine depending on the application.

In conclusion, the present study identifies bacterial compositional changes during typical poultry processing stages and before/after of antimicrobial treatments in the pilot processing plant obtained from microbiological cultivation as well as microbiome analysis and offers a comparison between the two methods. Investigation of the microbiota in chicken carcass rinsates at various processing stages was initiated in the present study and microbiome sequencing appears to be a viable approach for evaluating microbial composition of bacterial populations recovered from individual poultry carcasses. Also, the results of this study provide new insight into the antimicrobial treatments actually applicable to poultry processing plant to ensure poultry safety and may be of benefit to researchers and manufacturers.

## Methods

All experiments in the present study were conducted in accordance with relevant guidelines and regulations.

### Chicken processing stages

A total of 80 birds were randomly chosen and processed from raw bird to chicken carcass (stunner, bleeding tunnel, scalder, picker for depilation, hock cutter, evisceration, and chiller). PAA (Actrol, Zoetis, Florham Park, NJ) and Amplon (Zoetis) were prepared according to the manufacturer’s instruction. Antimicrobial treatments at four different locations to reduce bacterial populations on chicken were applied during processing including 1) washing with Amplon spray (pH1.3 and flow rate of 3 to 4 gpm using 4 × 1 gpm flood jet spray nozzles) after depilation (Fig. 1B), 2) simulated on-line reprocessing (OLR) with Amplon (pH 1.4 and chicken carcass after dipping in the Amplon solution for 15 s) after evisceration (Fig. 1C), 3) post-chilling with Amplon (pH 1.4 and 15 s dip) after main chilling (Fig. 1D), and 4) post-chilling with PAA (750 ppm and 15 s dip) after main chilling (Fig. 1D).

### Collection of chicken carcass rinsates

A University of Arkansas Institutional Animal Care and Use Committee (IACUC)-approved protocol was used to ensure humane treatment of the chickens (IACUC #15008). Chicken carcass rinsates were collected from 10 birds at each sampling point as shown in Fig. 1. To investigate microbial and microbiome analysis during poultry processing steps, each of the stepwise rinsate samples were collected from the chickens after the bleed out tunnel (group A), picker (group B), evisceration (group D), and chiller (group F) (Fig. 1A).

Washing with Amplon spray was applied after the picker (depilation) step. Rinsate samples were collected prior and post Amplon spray to assess bacterial population responses (group B and C in Fig. 1B). For the simulated OLR, before and after samples were collected (group D and E in Fig. 1C). To investigate the effect of two types of post-chilling, samples were collected from before post-chilling (group F in Fig. 1D), after chilling with Amplon (group G in Fig. 1D), and after chilling with PAA (group H in Fig. 1D).

A total of 80 chicken rinsates were utilized to determine Petrifilm aerobic plate counts (APCs), *Campylobacter* enumeration, and *Salmonella* prevalence as well as microbiome analysis. All birds were processed similarly and removed from their respective sampling points. Each chicken carcass was transferred to a sterile bag and 400 ml of sterile buffered peptone water (BPW; EMD Chemicals Inc., Gibbstown, NJ) were added. The sterile bags were manually shaken for 2 min assuring that all surfaces in- and exterior of the carcass were rinsed. Rinsates of ten birds from each group were obtained at the same time. Chicken carcass rinsates were transferred to a sterile bottle in the ice tray and immediately delivered to the laboratory within 10 min for microbial analyses.

### Experimental I: Microbiological analysis

Microbiological analysis of APC, *Campylobacter*, and *Salmonella* were performed based on the Microbiology Laboratory Guidebook (MLG) 3.02, 41.04, and 4.08 provided by United States Department of Agriculture-Food Safety and Inspection Service (USDA-FSIS) with some modification, respectively^41^.

### Petrifilm Aerobic plate count

One milliliter aliquots of chicken carcass rinsates were subjected to 10-fold serial dilutions up to 10^−6^ with 9 ml of sterile phosphate buffered saline (PBS; Amresco, Solon, OH). Each diluent (1 ml) was inoculated on the duplicate of APC Petrifilm (3 M Microbiology, St. Paul, MN) and incubated at 37 °C for 48 h. After incubation, total APCs were calculated based on the manufacturer’s instruction.

### *Campylobacter* enumeration

One milliliter of chicken carcass rinsates was spread onto four Campy-Cefex selective media (Acumedia, Lansing, MI) (250 μl each) and incubated at 42 °C for 48 h under microaerophilic conditions (GasPack Plus, Becton Dickinson and Company, Sparks, MD). *Campylobacter* suspected positive colonies were resuspended in the PBS for confirmation via PCR using a *Campylobacter* genus specific primer pair. Each PCR reaction included 2 μl of cell suspension, 500 nM of each primer (F: CAAGTTGCTACAATCTCAGCCA; R: GATAACACCATCTTTGCCCACT)^48^ and 10 μl of Jump Start Ready Mix (Sigma-Aldrich, St. Louis, MO), and distilled water up to 20 μl. PCR conditions were 94 °C for 5 min initially, followed by 34 cycles of 94 °C for 30 s, 60 °C for 30 s, 72 °C for 30 s. The final elongation step for 5 min at 72 °C was conducted at the end. Positive control with *C. jejuni* NCTC 11168 and negative control with water were also included in each reaction. PCR Amplicons were separated on a 1.5% agarose gel. Colonies which were successfully identified by PCR with 90 bp region of the targeted gene were recorded as *Campylobacter* positive.

### *Salmonella* isolation and identification

Each chicken carcass rinsate (20 ml) was transferred to 20 ml of BPW and incubated at 37 °C for 24 h. After incubation, pre-enriched sample (1 ml) was transferred to 9 ml of tetrathionate (TT) broth and incubated at 42 °C for 24 h. One loopful of the enrichment culture (10 μl) was streaked on brilliant green (BG) and xylose lysine agar tergitol 4 (XLT4) selective media (Becton Dickinson and Company) and incubated at 37 °C for 24 h. *Salmonella* suspected positive colonies were resuspended in the PBS for confirmation via PCR using a *Salmonella* subspecies I specific primer pair. Each PCR reaction contained 2 μl of suspected colony suspension, 500 nM of each primer (F: GGTGGCCTCGATGATTCCCG; R: CCCACTTGTAGCGAGCGCCG) targeting STM4057 gene^49^, 10 μl of Jump Start Ready Mix (Sigma-Aldrich), and distilled water up to 20 μl. PCR conditions consisted of an initial denaturation step of 94 °C for 5 min followed by 34 cycles of 94 °C for 30 s, 60 °C for 30 s, 72 °C for 30 s. Finally, samples were subjected to 72 °C for 5 min for elongation. Positive control with *S.* Typhimurium ATCC 14028 and negative control with water were also included in each reaction. The PCR amplicons were separated on the 1.5% agarose gel. Colonies which were successfully identified by PCR with the 137 bp region of the targeted gene were considered as *Salmonella* positive.

### Experimental 2: Microbiome analysis

Microbiome sequencing was performed via an Illumina MiSeq platform (Illumina, San Diego, CA) targeting the V4 hypervariable region of 16S rRNA following a detailed description in previously published research^20^.

### DNA extraction

#### DNA extraction from chicken rinsates

Each of the chicken rinsates (40 ml) collected from the eight processing stages (total 80 rinsates) were placed in a plastic 50-ml centrifuge tube and bacterial cells were harvested by centrifugation (Eppendorf centrifuge 5804R; Eppendorf, German) for 10 min at 11,000 rpm at 4 °C. The supernatant was discarded and the pellet was resuspended in 5 ml of sterile PBS. Genomic DNA was extracted using a QIAamp Fast DNA Stool Mini Kit (Qiagen, Valencia, CA) according to the manufacturer’s instruction.

#### DNA extraction from APC Petrifilm

Since no APCs were recovered in two samples in group H, the remaining 78 samples were subjected to microbiome analysis. The gel in the APC Petrifilm containing colonies was collected into sterile 2 ml microcentrifuge tubes. Genomic DNA was extracted using a Blood and Tissue Kit (Qiagen) according to the manufacturer instructions with some modification. Briefly, 400 μl enzymatic lysis buffer was added to the tube containing Petrifilm gel. After incubation at 37 °C for 30 min, 25 μl proteinase K and 200 μl Buffer AL was added to each tube and subsequently mixed by vortexing. After incubation at 56 °C for 30 min, the tubes were centrifuged at 14,000 rpm for 2 min and pink colored supernatant (approximately 200 to 250 μl) was transferred to a new 1.5 ml microcentrifuge tube. The next steps were the same as the instructions of the manufacturer.

#### DNA extraction from colonies on Campy-Cefex selective agar

Only plates having at least one *Campylobacter* suspected colony were used for sequencing analysis. No *Campylobacter* colonies were observed in 31 samples thus 49 samples were utilized for DNA isolation. All colonies on Campy-Cefex selective media were collected using a sterile loop and suspended in 2 ml of nuclease-free water. Genomic DNA was extracted using a DNeasy Blood and Tissue Kit (Qiagen) according to the manufacturer’s instructions.

The concentration and purity of all extracted DNA (DNA from chicken rinsates, APC Petrifilm, and colonies on Campy-Cefex selective media) was measured using a NanoDrop ND-1000 spectrophotometer (Thermo Fisher Scientific, Wilmington, DE) and subsequently diluted to 10 ng/μl with nuclease-free water and stored at −20 °C.

### Library preparation

The V4 region of 16S rRNA was amplified from each extracted genomic DNA from chicken rinsates, APC Petrifilm, and colonies on Campy-Cefex selective media using PCR with dual-index primers following previous research^20^. A mock community containing known sequences was utilized as a positive control.

### Sequencing via an Illumina MiSeq platform

A pooled library prepared with the method described in the section 2.4.2 and a PhiX control v3 were mixed with HT1 buffer and 0.2N fresh NaOH to yield the final concentration at 6 pM each. The resulting library and PhiX control v3 was combined to give a 95% 16S rRNA gene amplicon library and 5% PhiX control. A MiSeq^®^ v2 (500 cycle) Reagent cartridge (Illumina) was prepared according to the manufacturer’s instruction. Samples were sequenced using the Illumina MiSeq platform and sequencing procedures were monitored on the Illumina BaseSpace^®^ website.

### Sequencing data processing and analysis

Demultiplexed R1 and R2 sequencing read files were obtained from the Illumina BaseSpace website and Illumina reads were analyzed with the Quantitative Insights into Microbial Ecology (QIIME) pipeline (version 1.9.0). Paired-end reads were assembled with a script multiple_join_paired_ends.py and multiple_split_libraries_fastq.py. The assembled sequences were used to construct Operational Taxonomic Units (OTUs) with 97% identity and sequences were classified into the taxonomical levels based on the Greengenes 16S rRNA gene database. Chimeric OTUs were discarded and then OTUs were subsequently normalized. Alpha (rarefaction curve for OTUs, Chao1, and PD_Whole_Tree) and beta diversity were generated using weighted and unweighted UniFrac distance with a script core_diversity_analysis.py.

### Statistical analysis

The averages of plate counts were converted to log CFU/chicken. One way analysis of variance (ANOVA) was performed to compare differences of bacterial population or relative abundance among groups with JMP^®^ Genomics 7.0 (SAS Institute Inc., Cary, NC) at *P* < 0.05.

## Additional Information

**How to cite this article:** Kim, S. A. *et al*. Assessment of Chicken Carcass Microbiome Responses During Processing in the Presence of Commercial Antimicrobials Using a Next Generation Sequencing Approach. *Sci. Rep.* **7**, 43354; doi: 10.1038/srep43354 (2017).

**Publisher's note:** Springer Nature remains neutral with regard to jurisdictional claims in published maps and institutional affiliations.
